# Circulating free insulin-like growth factor-I and prostate cancer: a case-control study nested in the European prospective investigation into cancer and nutrition

**DOI:** 10.1186/s12885-023-11425-w

**Published:** 2024-06-03

**Authors:** Tuck Seng Cheng, Urwah Noor, Eleanor Watts, Michael Pollak, Ye Wang, James McKay, Joshua Atkins, Giovanna Masala, Maria-Jose Sánchez, Antonio Agudo, Jesús Castilla, Dagfinn Aune, Sandra M. Colorado-Yohar, Luca Manfredi, Marc J. Gunter, Valeria Pala, Andreas Josefsson, Timothy J. Key, Karl Smith-Byrne, Ruth C. Travis

**Affiliations:** 1 Cancer Epidemiology Unit, Nuffield Department of Population Health, University of Oxford, Richard Doll Building, Old Road Campus, Oxford, OX3 7LF UK; 2 Division of Cancer Epidemiology and Genetics, National Cancer Institute, Rockville, MD USA; 3 Oncology Department, McGill University and Segal Cancer Centre, Jewish General Hospital, Montreal, QC Canada; 4 Genomic Epidemiology Branch, International Agency for Research on Cancer, Lyon, France; 5 Cancer Risk Factors and Life-Style Epidemiology Unit, Institute for Cancer Research, Prevention and Clinical Network (ISPRO), Florence, Italy; 6 Escuela Andaluza de Salud Pública (EASP), Granada, 18011 Spain; 7 Instituto de Investigación Biosanitaria ibs.GRANADA, Granada, 18012 Spain; 8 Centro de Investigación Biomédica en Red de Epidemiología y Salud Pública (CIBERESP), Madrid, 28029 Spain; 9 Department of Preventive Medicine and Public Health, University of Granada, Granada, 18071 Spain; 10 Unit of Nutrition and Cancer, Catalan Institute of Oncology - ICO, L’Hospitalet de Llobregat, Barcelona, Spain; 11 Nutrition and Cancer Group; Epidemiology, Public Health, Cancer Prevention and Palliative Care Program; Bellvitge Biomedical Research Institute - IDIBELL, L’Hospitalet de Llobregat, Barcelona, Spain; 12 Instituto de Salud Pública de Navarra – IdiSNA, Pamplona, Spain; 13 CIBER of Epidemiology and Public Health (CIBERESP), Madrid, Spain; 14 Department of Epidemiology and Biostatistics, School of Public Health, Imperial College London, London, UK; 15 Department of Nutrition, Oslo New University College, Oslo, Norway; 16 Department of Endocrinology, Morbid Obesity and Preventive Medicine, Oslo University Hospital Ullevå, Oslo, Norway; 17 Department of Epidemiology, Murcia Regional Health Council-IMIB, Murcia, Spain; 18 CIBER Epidemiología y Salud Pública (CIBERESP), Madrid, Spain; 19 Research Group on Demography and Health, National Faculty of Public Health, University of Antioquia, Medellín, Colombia; 20 Centre for Biostatistics, Epidemiology, and Public Health (C-BEPH), Department of Clinical and Biological Sciences, University of Turin, Orbassano, TO Italy; 21 Nutrition and Metabolism Branch, International Agency for Research on Cancer (IARC- WHO), Lyon, France; 22 Epidemiology and Prevention Unit, Department of Research, Fondazione IRCCS Istituto Nazionale dei Tumori, Milan, Italy; 23 Department of Urology, Institute of Clinical Sciences, Sahlgrenska Academy, University of Gothenburg, Gothenburg, Sweden; 24 Wallenberg Center for Molecular Medicin, Umeå University, Umeå, Sweden; 25 Department of Urology and Andrology, Institute of surgery and perioperative Sciences, Umeå University, Umeå, Sweden

**Keywords:** Free IGF-1, Prostate cancer, Histological grade, Tumor stage, Aggressiveness

## Abstract

**Background:**

Circulating total insulin-like growth factor-I (IGF-I) is an established risk factor for prostate cancer. However, only a small proportion of circulating IGF-I is free or readily dissociable from IGF-binding proteins (its bioavailable form), and few studies have investigated the association of circulating free IGF-I with prostate cancer risk.

**Methods:**

We analyzed data from 767 prostate cancer cases and 767 matched controls nested within the European Prospective Investigation into Cancer and Nutrition cohort, with an average of 14-years (interquartile range = 2.9) follow-up. Matching variables were study center, length of follow-up, age, and time of day and fasting duration at blood collection. Circulating free IGF-I concentration was measured in serum samples collected at recruitment visit (mean age 55 years old; standard deviation = 7.1) using an enzyme-linked immunosorbent assay (ELISA). Conditional logistic regressions were performed to examine the associations of free IGF-I with risk of prostate cancer overall and subdivided by time to diagnosis (≤ 14 and > 14 years), and tumor characteristics.

**Results:**

Circulating free IGF-I concentrations (in fourths and as a continuous variable) were not associated with prostate cancer risk overall (odds ratio [OR] = 1.00 per 0.1 nmol/L increment, 95% CI: 0.99, 1.02) or by time to diagnosis, or with prostate cancer subtypes, including tumor stage and histological grade.

**Conclusions:**

Estimated circulating free IGF-I was not associated with prostate cancer risk. Further research may consider other assay methods that estimate bioavailable IGF-I to provide more insight into the well-substantiated association between circulating total IGF-I and subsequent prostate cancer risk.

**Supplementary Information:**

The online version contains supplementary material available at 10.1186/s12885-023-11425-w.

## Background

Prostate cancer is the second most commonly diagnosed cancer and the fifth leading cause of cancer deaths among men worldwide [1]. The global incidence and mortality of prostate cancer are predicted to increase by 80% and to almost double by 2040, respectively [2, 3]. Higher circulating insulin-like growth factor-I (IGF-I) concentration is one of a very small number of established etiological risk factors for prostate cancer. Evidence in favor of a relationship between IGF-I and prostate cancer risk stems principally from a consistent risk association identified in several large prospective observational studies [4, 5]. More recently, additional evidence from both Mendelian randomization (MR) and *cis* colocalization analyses have identified a shared genetic cause between circulating IGF-I concentrations and prostate cancer risk at the *IGFI* locus [6, 7].

IGF-I is a growth-promoting peptide hormone, which, following the binding to its cognate receptor, stimulates cell proliferation and survival and decreases apoptosis, thereby increasing the risk of carcinogenesis [8, 9]. IGF-I is produced mainly by the liver (~ 75%) and also locally in many tissues [9]. In the blood circulation, IGF-I is predominantly bound to IGF binding proteins (IGFBPs), particularly IGFBP-3 (accounting for 75–80% of bound IGF-I) [10]. Only a small portion of circulating IGF-I (~ 1%) is free (or unbound) or readily dissociable from IGFBPs [11, 12]. This fraction is hypothesized to be the most bioactive and more readily available to bind to IGF-I receptors on cell surfaces to activate intracellular signaling cascades [13, 14].

The role of IGF-I in prostate cancer risk has been well-characterized from studies of circulating total IGF-I, which includes both bound and free IGF-I [4–7, 15]. To our knowledge, however, only three prospective studies of prostate cancer risk have measured circulating free IGF-I concentration using immunoradiometric assay (IRMA) [16] or enzyme-linked immunosorbent assay (ELISA) [17, 18], with sample sizes up to 1076 men; they did not identify associations but may not have had the power to detect small to moderate associations.

The present study aimed to examine the association between circulating free IGF-I and subsequent risk of prostate cancer in a case-control study nested within the European Prospective Investigation into Cancer and Nutrition (EPIC) cohort. Using a recently developed ELISA for free IGF-I [19], we measured free IGF-I levels in baseline serum samples from men who subsequently developed prostate cancer and their matched controls [median time to diagnosis: 14 years, interquartile range (IQR) = 2.9] in a large sample (767 pairs or n = 1534). We assessed the association of free IGF-1 with prostate cancer risk overall and by time to diagnosis, and also with risk by tumor subtypes according to histological grade, tumor stage, and aggressiveness.

## Methods

### Study population

EPIC is a large prospective multicenter cohort study that recruited more than 521,000 participants (153,426 men), who were predominantly white and aged 35–70 years old, from 23 centers in 10 European countries (Denmark, France, Germany, Greece, Italy, the Netherlands, Norway, Spain, Sweden and the United Kingdom) [20]. At the recruitment visit between 1992 and 1999, information on diet, lifestyle, medical history, and anthropometric measurements were recorded. Fasting or non-fasting blood samples were also collected from 387,889 individuals (137,000 men), and are stored at local centers and at the International Agency for Research on Cancer – World Health Organization (IARC-WHO) in Lyon, France. The current study included male participants with baseline blood samples from Germany, Italy, the Netherlands, Spain and the United Kingdom, which were collected according to a standardized protocol. Plasma, serum, erythrocytes, and buffy coat were separated by centrifugation and aliquoted into 28 straws for storage, until required for laboratory analysis. All of the serum samples for this study were thawed for removal from storage straws and refrozen, and then thawed again for aliquotting in preparation for assaying.

### Ascertainment of prostate cancer cases and controls

Information on cancer incidence, tumor characteristics, vital status, and cause of death was ascertained through population-based cancer registries in Italy, the Netherlands, Spain and the United Kingdom, and by active follow-up with different sources in Germany, including health insurance records, municipality registries, and hospital- or physician-based cancer and pathology registries. Prostate cancer cases were identified as men diagnosed with first incident prostate cancer based on the International Classification of Diseases 10th revision code (ICD-10: C61) [21], after blood collection and before the end of follow-up in 2013 (i.e. fourth round of EPIC endpoint follow-up, known as phase 4). These cases were matched one-to-one with controls who were randomly selected among male cohort participants who were free of cancer (excluding non-melanoma skin cancer) and alive at the time of diagnosis of the index case, using an incidence density sampling protocol. Matching variables were study center, length of follow-up (± 6 months), age at blood collection (± 6 months), time at blood collection (± 1 h) and fasting duration at blood collection (< 3 h, 3–6 h or > 6 h). The present analyses included 767 cases with 767 matched controls.

Information on histological grade and tumor stage at diagnosis was available for 641 (83.6%) cases and 406 (52.9%), respectively. For histological grade, there were 545 low-intermediate grade (Gleason score < 8, or grade coded in the recruitment center as well, moderately or poorly differentiated) and 96 high grade (Gleason score ≥ 8, or grade coded in the recruitment center as undifferentiated). For tumor stage, 273 cases were clinically localized (tumor-node-metastasis (TNM) staging score of T0-T2 and N0/Nx and M0, or stage coded in the recruitment center as localized) and 134 cases were clinically advanced (T3-T4 and/or N1-N3 and/or M1, or stage coded in the recruitment center as metastasis). Death from prostate cancer (n = 38) was defined as prostate cancer recorded as the underlying cause on the death certificate. We also further classified aggressive prostate cancer (n = 229) as those which were clinically advanced and/or high grade and/or prostate-specific antigen (PSA) > 20 ng/ml at diagnosis based on the definition from the European Association of Urology [22], and/or those who died from prostate cancer.

### Measurement of circulating free IGF-I and other analytes

Serum free IGF-I concentrations were assayed, with blinding to case-control status, in the laboratory of Dr. Michael Pollak at McGill University in Montreal, Canada, using ELISA (Ansh Labs, Webster, TX, USA) in 2021–2022. This assay is referred to as a highly sensitive two-site or “sandwich” method that directly detects free IGF-I that is bound between the first capture antibody immobilized on the microtiter plate and the second detection antibody specific for free IGF-I [11, 19]. Bound IGF-I is not detected since the epitope of IGF-I in IGF-I/IGFBPs complexes is concealed. Free IGF-I was measured in duplicate for each sample and mean values were used for analyses. All measures below the lower limit of detection (LOD, 0.33 ng/mL) (n = 525, 34%) were set to be 0.165 ng/mL, which is the midpoint between 0 and the LOD. The inter- and intra-batch coefficients of variation for this assay were 4.73% and 1.49%, respectively.

Measurements of serum total IGF-I, IGFBP-1, IGFBP-2, IGFBP-3, IGF-II, testosterone and sex hormone binding globulin (SHBG) concentrations were performed using ELISA or electrochemiluminescence immunoassay, as described in detail elsewhere [23, 24]. Total IGF-I was assayed with the elimination of IGFBPs using an acid ethanol precipitation step. Free testosterone concentrations were estimated using a formula based on the law mass action from measured total testosterone and SHBG concentrations [25, 26], assuming a constant albumin concentration of 43 g/L [23].

### Statistical analyses

Differences in selected participant characteristics between prostate cancer cases and controls were compared using chi-squared tests for categorical variables and t-tests for continuous variables. Given the left truncated distribution for free IGF-I owing to concentrations below the LOD, correlations between free IGF-I and other members of the IGF axis analytes (IGFBP-1, IGFBP-2, IGFBP-3, IGF-II) and sex hormones (testosterone, free testosterone, SHBG) were estimated using Spearman’s rank tests. Differences in free IGF-I concentrations by categories of selected participant characteristics among controls and cases were assessed using analysis of covariance, adjusted for laboratory batch, and age at blood collection, body mass index (BMI) and/or recruitment center. The concentrations of free IGF-I and other IGF axis analytes were presented as geometric means with 95% confidence interval (CI).

The association between circulating free IGF-I concentrations and overall prostate cancer risk was estimated using logistic regression models conditioned on the matching factors and adjusted for laboratory batch. Circulating free IGF-I concentrations were modelled in fourths (based on quartile cut-points defined among controls) (Table 1), and as a continuous variable. Linear trends for the associations of free IGF-I with risk were calculated across the medians within each fourth of free IGF-I. These analyses were repeated by time to diagnosis (≤ 14 and > 14 years), fasting status and BMI (< 30 kg/m^2^ and ≥ 30 kg/m^2^) as well as by tumor subtype of histological grade, tumor stage and aggressiveness. Similarly, the associations of circulating total IGF-I concentrations with risks for overall and aggressive prostate cancer were tested.

**Table 1:** Odds ratios (95% confidence intervals) for circulating free and total IGF-I concentrations in relation to risks for overall prostate cancer and prostate cancer by subtype

		Q1	Q2	Q3	Q4	P for trend	Continuous	P value
**Free IGF-I**	Median (nmol/L) (range)	0.022	0.073 (0.043–0.098)	0.132 (> 0.098–0.188)	0.319 (> 0.188–7.718)		per 0.1 nmol/L increase	
*Overall prostate cancer*	Cases/controls, n	260/265	176/168	159/167	172/167		767/767	
	OR (95% CI)^a^	1.00	1.07 (0.81, 1.41)	0.97 (0.73, 1.30)	1.05 (0.78, 1.42)	0.806	1.00 (0.99, 1.02)	0.892
*Overall prostate cancer* by time to diagnosis								
≤ 14 years	Cases/controls, n	136/123	80/84	72/86	78/73		366/366	
	OR (95% CI)^a^	1.00	0.82 (0.54, 1.25)	0.74 (0.48, 1.14)	0.94 (0.60, 1.47)	0.879	0.98 (0.96, 1.01)	0.207
> 14 years	Cases/controls, n	124/142	96/84	87/81	94/94		401/401	
	OR (95% CI)^a^	1.00	1.32 (0.90, 1.93)	1.25 (0.84, 1.87)	1.16 (0.78, 1.73)	0.666	1.01 (0.99, 1.04)	0.189
*Histological grade*								
Low-intermediate	Cases/controls, n	193/189	125/125	115/120	112/111		545/545	
	OR (95% CI)^a^	1.00	0.98 (0.70, 1.37)	0.92 (0.65, 1.30)	0.99 (0.69, 1.42)	0.966	1.00 (0.98, 1.02)	0.848
High grade	Cases/controls, n	30/38	22/18	18/21	26/19		96/96	
	OR (95% CI)^a^	1.00	1.42 (0.65, 3.08)	1.12 (0.51, 2.46)	1.79 (0.75, 4.27)	0.213	1.00 (0.97, 1.04)	0.802
*Tumor stage*								
Localized	Cases/controls, n	98/100	65/69	61/54	49/50		273/273	
	OR (95% CI)^a^	1.00	0.97 (0.62, 1.51)	1.17 (0.72, 1.89)	1.01 (0.61, 1.65)	0.904	1.01 (0.98, 1.04)	0.624
Advanced	Cases/controls, n	44/40	37/29	25/36	27/28		134/134	
	OR (95% CI)^a^	1.00	1.15 (0.60, 2.22)	0.64 (0.33, 1.25)	0.90 (0.43, 1.88)	0.503	0.97 (0.92, 1.02)	0.208
*Aggressiveness*								
Aggressive/fatal	Cases/controls, n	76/72	57/55	39/56	57/47		229/229	
	OR (95% CI)^a^	1.00	0.98 (0.61, 1.59)	0.68 (0.40, 1.15)	1.19 (0.68, 2.07)	0.651	0.99 (0.96, 1.01)	0.325
**Total IGF-I**	Median (nmol/L) (range)	14.19 (6.18–16.03)	17.64 (16.04–18.98)	20.27 (18.99–21.74)	24.38 (21.75–57.68)		per 5 nmol/L increase	
*Overall prostate cancer*	Cases/controls, n	145/180^c^	180/177	176/178	212/178		713/713	
	OR (95% CI)	1.00	1.27 (0.94, 1.73)	1.23 (0.91, 1.68)	1.50 (1.11, 2.02)	0.014	1.18 (1.05, 1.33)	0.005
*Aggressiveness*								
Aggressive/fatal	Cases/controls, n	46/56	58/50	50/60	59/47		214/214	
	OR (95% CI)^a^	1.00	1.42 (0.83, 2.44)	1.01 (0.58, 1.75)	1.55 (0.89, 2.71)	0.246	1.25 (1.00, 1.56)	0.051

^a^model conditioned on the matching variables: center, follow-up time, fasting status, age at blood collection and time at blood collection, and adjusted for laboratory batch (only for free IGF-I)

All statistical analyses were conducted using Stata 17.0 (Stata Corp LP, College Station, TX). Findings were plotted using “ggplot2” package in R 4.1.1.

## Results

### Participants’ characteristics

The present analyses included 767 incident prostate cancer cases and 767 matched controls, with mean age at blood collection of 55 (standard deviation = 7.1) years old. For cases, the mean age at diagnosis was 69 years and the median time from blood collection to diagnosis was 14 (IQR = 2.9) years. No material differences in selected characteristics were found between men who developed prostate cancer and men who did not (Table 2).

**Table 2:** Characteristics of 767 men who developed prostate cancer and 767 matched control participants in EPIC

Demographic and lifestyle characteristics	Cases (n = 767)	Controls (n = 767)	P values
Age at blood collection, years (SD)	54.6 (7.1)	54.6 (7.1)	
Weight, kg (SD)^1^	79.4 (10.5)	80.1 (11.0)	0.209
Height, cm (SD)^1^	171.9 (7.1)	171.7 (7.2)	0.558
Body mass index, kg/m^2^ (SD)^1^	26.9 (3.2)	27.2 (3.6)	0.074
Country, n (%)			
Germany	37 (4.8)	37 (4.8)	
Italy	178 (23.2)	178 (23.2)	
Spain	292 (38.1)	292 (38.1)	
The Netherlands	50 (6.5)	50 (6.5)	
UK	210 (27.4)	210 (27.4)	
Education level, n (%)^1^			0.842
None/primary	337 (46.5)	337 (46.0)	
Secondary	246 (33.9)	258 (35.3)	
Tertiary	142 (19.6)	137 (18.7)	
Smoking status, n (%)^1^			0.498
Never	262 (34.7)	244 (32.0)	
Previous	294 (38.9)	302 (39.6)	
Current	199 (26.4)	216 (28.4)	
Alcohol consumption, n (%)^1^			0.945
≤ 9 g/day	288 (38.4)	296 (38.6)	
10–19 g/day	128 (17.1)	137 (17.9)	
20–39 g/day	179 (23.9)	184 (24.0)	
≥ 40 g/day	155 (20.7)	150 (19.6)	
Diabetes status, n (%)^1^			1.000
No	741 (96.9)	741 (96.9)	
Yes	24 (3.1)	24 (3.1)	
**Cases only**			
Age at diagnosis, years (SD)	68.7 (7.2)		
Time from blood collection to diagnosis, years (SD)	14.1 (2.1)		
Prostate-specific antigen at diagnosis, n (%)^1^			
< 3 ng/ml	10 (2.0)		
3-<10 ng/ml	298 (59.0)		
10-<50 ng/ml	167 (33.1)		
≥ 50 ng/ml	30 (5.9)		
Grade of disease, n (%)^1,2^			
Low-intermediate	545 (85.0)		
High	96 (15.0)		
Stage of disease, n (%)^1,3^			
Localised	273 (67.2)		
Advanced	133 (32.8)		
Aggressiveness, n (%)^1,4^			
Non-aggressive	183 (44.4)		
Aggressive/fatal	229 (55.6)		
Death from prostate cancer, n (%)^5^	38 (5.0)		

^1^Unknown values for some participants (n = 3-361); the calculations of percentages exclude missing values

^2^Gleason score < 8 or coded as well, moderately or poorly differentiated for low-intermediate grade and Gleason score ≥ 8 or coded as undifferentiated for high grade

^3^The tumor-node-metastasis (TNM) system was used to categorize stages of prostate cancer; localized:≤T2 and N0/x and M0,or coded as localized; advanced: T3–4 and/or N1–3 and/or M1, or coded as metastasis;

^4^Non-aggressive: ≤T2 or coded as localised, Gleason score < 8 or coded as well, moderately or poorly differentiated and prostate-specific antigen ≤ 20 ng/ml; and aggressive:T3-T4 or coded as metastasis, and/or Gleason score ≥ 8 or coded as undifferentiated and/or prostate-specific antigen > 20 ng/ml and/or death from prostate cancer

^5^Prostate cancer listed as the underlying cause of death on the death certificate during follow-up

Total IGF-I concentration was higher in cases than controls (geometric means = 19.2 nmol/L, 95% CI: 18.89–19.57 vs. 18.64, 18.32–18.98), while no differences in free IGF, IGFBPs or IGF-II concentrations were observed (Supplemental Table 1). Overall, free IGF-I concentration was modestly positively correlated with total IGF-I concentration (Spearman’s rank correlation coefficient, ρ = 0.230) and the ratio of total IGF-I to IGFBP-3 (ρ = 0.250), but not with other IGFBPs, IGF-II or sex hormone concentrations (Supplementary Tables 2 and Supplementary Fig. 1).

Table 3 shows the differences in free IGF-I concentrations across selected characteristics among controls and cases. Men who had higher BMI, blood collected at an earlier time of day, higher alcohol consumption, who fasted before blood collection, or who were current smokers tended to have lower free IGF-I concentration, in both controls and cases.

**Table 3:** Adjusted geometric mean free IGF-I concentration across characteristics in 767 controls and 767 prostate cancer cases

Characteristics		Controls (n = 767)			Cases (n = 767)			
	N	Free IGF-I (nmol/L)			Free IGF-I (nmol/L)			
		Mean (95% CI)	P value	N		Mean (95% CI)		P value
Age at blood collection (years)^b^								
< 55	398	0.08 (0.07, 0.09)		399		0.09 (0.08, 0.10)		
55–59	198	0.08 (0.07, 0.10)		196		0.07 (0.06, 0.08)		
60–64	114	0.08 (0.06, 0.10)		114		0.07 (0.06, 0.09)		
65–69	42	0.08 (0.06, 0.12)		43		0.10 (0.07, 0.14)		
≥ 70	15	0.06 (0.03, 0.11)	0.918	15		0.08 (0.04, 0.14)		0.270
Height (cm)^c^								
≤ 170	329	0.08 (0.07, 0.09)		319		0.07 (0.06, 0.08)		
171–175	206	0.07 (0.06, 0.08)		191		0.09 (0.08, 0.11)		
176–180	136	0.09 (0.07, 0.11)		161		0.08 (0.07, 0.09)		
> 180	96	0.09 (0.07, 0.11)	0.290	96		0.10 (0.07, 0.12)		0.054
Body mass index (kg/m^2^)^c^								
< 22.5	58	0.10 (0.07, 0.14)		63		0.11 (0.08, 0.15)		
22.5–24.9	144	0.09 (0.07, 0.11)		155		0.09 (0.07, 0.11)		
25-27.4	247	0.08 (0.07, 0.10)		250		0.09 (0.07, 0.10)		
27.5–29.9	173	0.08 (0.07, 0.10)		180		0.06 (0.05, 0.07)		
≥ 30	145	0.06 (0.05, 0.08)	0.040	119		0.08 (0.06, 0.10)		0.005
Country^d^								
Germany	37	0.07 (0.05, 0.10)		37		0.05 (0.04, 0.08)		
Italy	178	0.06 (0.05, 0.07)		178		0.06 (0.05, 0.07)		
Spain	292	0.07 (0.06, 0.08)		292		0.08 (0.07, 0.09)		
The Netherlands	50	0.09 (0.07, 0.13)		50		0.07 (0.05, 0.10)		
UK	210	0.11 (0.10, 0.13)	< 0.001	210		0.12 (0.10, 0.14)		< 0.001
Time at blood collection^a,e^								
00:00–09:59	345	0.07 (0.06, 0.08)		353		0.07 (0.06, 0.08)		
10:00–12:59	208	0.09 (0.08, 0.10)		192		0.09 (0.07, 0.10)		
13:00–23:59	192	0.09 (0.08, 0.11)	0.012	200		0.10 (0.08, 0.12)		0.027
Fasting status^a,e^								
No	411	0.09 (0.08, 0.10)		411		0.09 (0.08, 0.10)		
Yes	334	0.07 (0.06, 0.08)	0.003	334		0.07 (0.06, 0.08)		0.005
Duration between last meal and blood collection^a,e^								
< 3 h	278	0.09 (0.07, 0.10)		278		0.08 (0.07, 0.10)		
3–6 h	133	0.10 (0.08, 0.12)		133		0.11 (0.09, 0.13)		
> 6 h	334	0.07 (0.06, 0.08)	0.005	334		0.07 (0.06, 0.08)		0.002
Smoking status^a,e^								
Never	244	0.09 (0.08, 0.11)		262		0.09 (0.08, 0.11)		
Previous	302	0.09 (0.08, 0.10)		294		0.08 (0.07, 0.10)		
Current	216	0.06 (0.05, 0.07)	< 0.001	199		0.07 (0.06, 0.08)		0.039
Alcohol consumption (g/day)^e^								
≤ 9	296	0.08 (0.07, 0.10)		288		0.09 (0.08, 0.11)		
10–19	137	0.09 (0.08, 0.11)		128		0.10 (0.08, 0.12)		
20–39	184	0.08 (0.07, 0.09)		179		0.07 (0.06, 0.08)		
≥ 40	150	0.06 (0.05, 0.08)	0.046	155		0.07 (0.05, 0.08)		0.001
Education level^a,e^								
None/primary	337	0.07 (0.06, 0.08)		337		0.07 (0.06, 0.08)		
Secondary	258	0.09 (0.08, 0.10)		246		0.08 (0.07, 0.09)		
Tertiary	137	0.08 (0.07, 0.10)	0.063	142		0.10 (0.08, 0.13)		0.013
Diabetes status^a,e^								
No	742	0.08 (0.07, 0.09)		741		0.08 (0.07, 0.09)		
Yes	24	0.06 (0.04, 0.10)	0.289	24		0.08 (0.05, 0.13)		0.923

^a^Unknown values for some participants (n = 1–35)

^b^adjusted for recruitment centre and batch

^c^adjusted for age at blood collection, recruitment centre and batch

^d^adjusted for age at blood collection, body mass index and batch

^e^adjusted for age at blood collection, body mass index, recruitment centre and batch

### IGF-I and prostate cancer

**Fig. 1:**
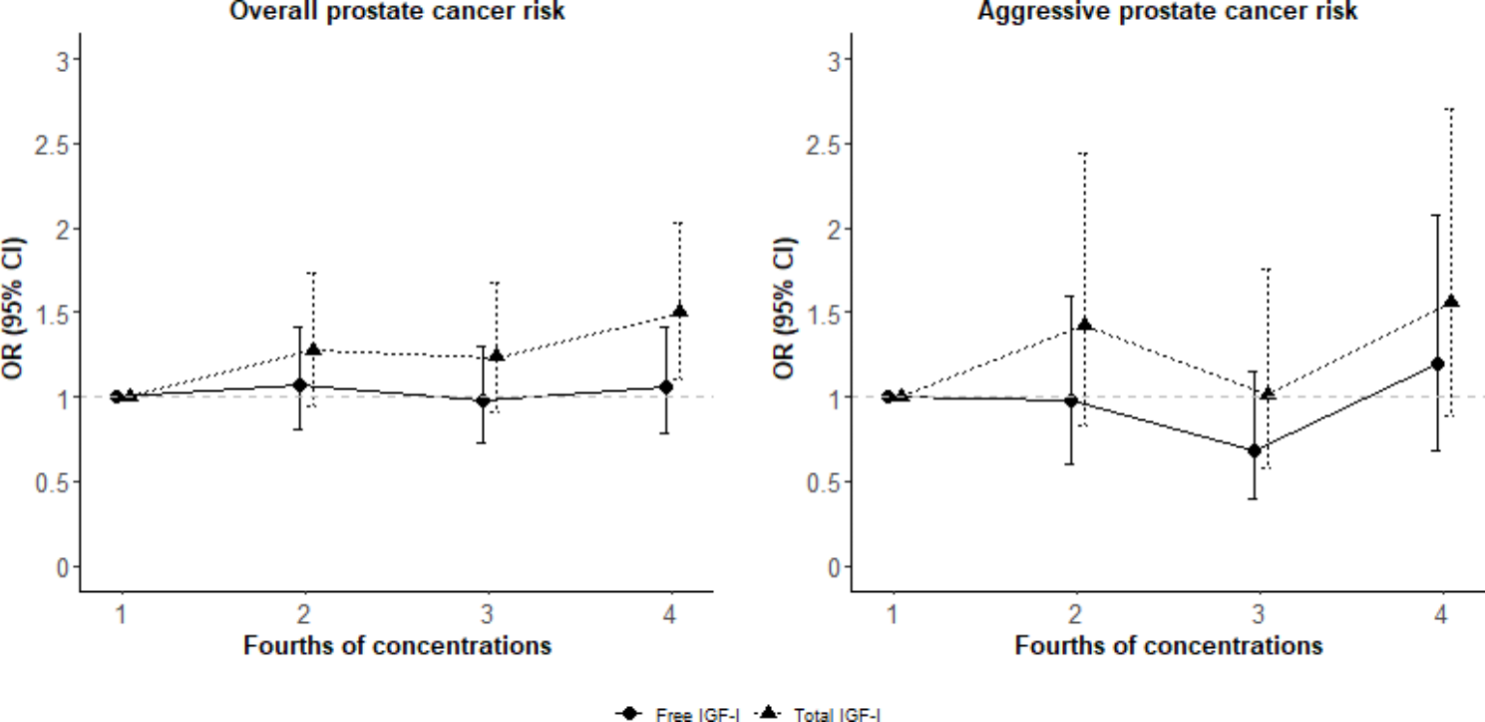
**Adjusted associations of circulating free and total IGF-I concentrations with overall and aggressive prostate cancer risks in EPIC** Models conditioned on the matching variables: center, follow-up time, fasting status, age at blood collection and time at blood collection, and adjusted for laboratory batch (only for free IGF-I)

Figure 1; Table 1 show the adjusted associations of circulating free and total IGF-I concentrations with prostate cancer risk. Higher free IGF-I concentration (in fourths or as a continuous variable) was not associated with total prostate cancer risk [Odds ratio (OR) = 1.00 per 0.1 nmol/L increase, 95% CI: 0.99, 1.02]. Similarly, there were no significant associations between free IGF-I concentration and total prostate cancer risk when stratified by time to diagnosis, fasting status and BMI (< 30 kg/m^2^ and ≥ 30 kg/m^2^) (Supplementary Table 3). Also, higher free IGF-1 concentration was not associated with prostate cancer risk when analyses were repeated by histological grade, tumor stage or aggressiveness. Higher circulating total IGF-I concentration was associated with higher overall (OR = 1.18 per 5 nmol/L increase, 95% CI: 1.05, 1.33) and possibly aggressive prostate cancer risks (OR = 1.25, 95% CI: 1.00, 1.56).

## Discussion

In this matched nested case-control study with a long duration of follow-up from blood collection among European men, we found no association of circulating free IGF-I concentration measured using a recently developed ELISA with prostate cancer risk, and these findings did not vary by time to diagnosis or tumor subtype. In contrast, we observed a positive association of total IGF-I concentration with total prostate cancer risk, as we and others have previously reported in EPIC and other studies [4–7, 15].

Our null findings for free IGF-I are consistent with the two previous nested case-control studies of free IGF-I and prostate cancer [16, 17]. In the Physician’s Health Study (PHS), Mucci et al. measured fasting or non-fasting plasma free IGF-I concentration in 545 matched case-control pairs using a different ELISA and found no association with prostate cancer risk [17]. In the European Randomized Study of Screening for Prostate cancer, Janssen et al. used IRMA to assay serum free IGF-I (201 pairs) and found no difference in free IGF-I concentrations between prostate cancer cases and matched controls [16]. In addition to these studies of overall prostate cancer, in a case-only study in the PHS and Health Professionals Follow-Up Study, Ma et al. found no evidence for an association of free IGF-I with risk of lethal compared to nonlethal prostate cancer (524 nonlethal, 434 lethal cases) [18].

Higher circulating total IGF-I concentration is one of a limited number of established causal and potentially modifiable risk factors for prostate cancer risk, with strong evidence from both large prospective observational and genetic study designs [4–7, 15]. Higher circulating free IGF-I, which may reflect the bioactive form of total IGF-I, has been suggested as one possible mechanism driving the observed association of total IGF-I with prostate cancer risk. Previous studies have identified cancer-promoting properties for free-IGF-I, including mitotic and antiapoptotic effects [27], motivating the present study. Nevertheless, we did not observe an association between circulating free IGF-I concentration and prostate cancer, despite the positive association for circulating total IGF-I concentration in the same sample.

Our null findings for circulating free IGF-I may relate to the complexity of IGF signaling; IGFBPs can both enhance and inhibit IGF-I signaling [9]. The prostate also produces IGFs, IGFBPs and IGFBP proteases locally [10, 28]. Therefore, circulating free IGF-I may not be a good predictor of intra-prostatic IGF-I bioactivity. While circulating free IGF-I concentration might not be relevant for prostate cancer risk, this does not exclude the possible biological effect of free IGF-I in prostate tissue on prostate cancer development. Future studies using assays that quantify IGF1 receptor activation or expression in prostate tissue may help to further understand relationships between IGF-I signaling and prostate cancer.

Our null results for free but not total IGF-I might reflect the inherent difficulty in measuring free IGF-I due to its short half-life (1–2 min) in the circulation, in contrast to circulating total IGF-I (up to 24 h) [14]. It is also possible that IGF-I released from the IGFBP-bound IGF-I complex immediately binds to its receptor once IGFBPs are cleaved or bind to the target cell surface [10], and thus this bioavailable IGF-I cannot be well estimated based on free IGF-I in blood samples. While about one-third of participants had free IGF-I below the LOD, coefficients of variation were low and duplicate measurements were very highly correlated, implying that measurement error in between-individual variations of current assay is likely to be modest. Additionally, we observed a modest but highly significant positive correlation for free IGF-I with total IGF-I, consistent with a previous study [17].

Several limitations in the present analyses need to be acknowledged. Although we included a large sample in the overall analyses, the statistical power and thus our ability to detect associations in the stratified analyses was more limited. The number of prostate cancer deaths was also modest. Levels of circulating free IGF-I concentrations might have been affected by storage time since the serum samples used for IGF-I measurements in 2021–2022 had been stored since recruitment in the 1990s, nonetheless we observed associations for total IGF-I concentrations using the same samples. Also, the estimate of free IGF-I in this study includes both free and readily dissociable IGF-I [11, 19]; however, readily dissociable IGF-I, unlike stably bound IGF-I, may also have biological relevance. Additionally, our study considered free IGF-I concentration for each individual measured only at a single timepoint. Given the low proportion of free IGF-I in circulation (~ 1%), even modest measurement error may induce considerable attenuation of risk estimates where single measurements may not adequately capture average concentrations over the medium to long term. Furthermore, we measured free IGF-I concentration in circulating blood samples. Previous studies have suggested there may be effects of locally accumulated free IGF-I in tissues on risk for prostate cancer [27, 29], which we did not estimate in the present study. Although participants in the present study had lower concentrations in IGF axis analytes than other studies, the magnitude of the association between circulating total IGF-I concentration and prostate cancer was similar across studies [4–7]. Finally, our study analyzed white men, and thus our findings may not be generalizable to other populations.

## Conclusions

In conclusion, this study did not find evidence of an association of higher circulating free IGF-I, measured using a recently developed sandwich ELISA, with subsequent risk of prostate cancer overall or by follow-up duration and prostate tumor characteristics including histological grade, tumor stage and aggressiveness. Further research may consider other assays that estimate the bioavailability of circulating IGF-I, as well as methods for measuring free IGF-I in prostate tissue, to deepen the understanding of potential pathways and mechanisms for the substantiated association between circulating total IGF-I and subsequent prostate cancer development and progression.

### Electronic supplementary material

Below is the link to the electronic supplementary material.

Supplementary Material 1

## Data Availability

For information on how to submit an application for gaining access to EPIC data, please follow the instructions at http://epic.iarc.fr/access/index.php.

## Declarations

### Abbreviations

BMI body mass index.

CI confidence interval.

ELISA enzyme-linked immunosorbent assay.

EPIC European Prospective Investigation into Cancer and Nutrition.

IGFBP IGF binding protein.

IGF-I insulin-like growth factor-I.

IRMA immunoradiometric assay.

IQR interquartile range.

LOD limit of detection.

MR Mendelian randomization.

PSA prostate-specific antigen.

SHBG sex hormone binding globulin.

TNM tumor-node-metastasis.
