# Supplementary material for: Circulating free insulin-like growth factor-I and prostate cancer: a case-control study nested in the European prospective investigation into cancer and nutrition
Source: BMC Cancer. 2024 Jun 3;24:676. doi: 10.1186/s12885-023-11425-w (PMC11145848; doi:10.1186/s12885-023-11425-w)
Supplement: Supplementary file 1 — Supplementary Material 1 [file 12885_2023_11425_MOESM1_ESM.docx]

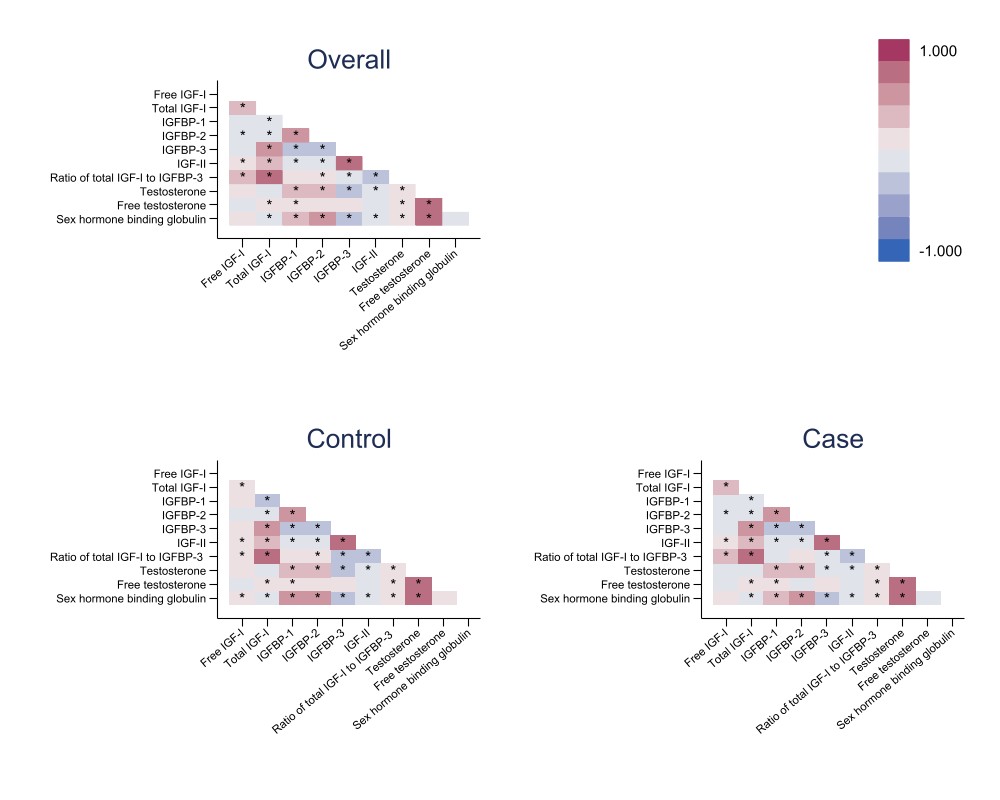
**Supplemental Figure 1: Spearman’s rank correlations between IGF axis analytes and sex hormone concentrations**

* indicates P value <0.05

| **Supplementary Table 1: Geometric means (95% confidence intervals) of IGF-axis analytes and sex hormone concentrations for 767 men who developed prostate cancer and 767 matched controls in EPIC** | | | |  |  |
| --- | --- | --- | --- | --- | --- |
|  | **Geometric mean (95% confidence interval)** | | **P value** | | |
|  | **Cases** | **Controls** |  | | |
| **IGF-axis analytes** |  |  |  | | |
| Free IGF-I (nmol/L) | 0.08 (0.07, 0.09) | 0.08 (0.07, 0.09) | 0.780 | | |
| Total IGF-I (nmol/L)^a^ | 19.22 (18.89, 19.57) | 18.64 (18.32, 18.98) | 0.017 | | |
| Percentage of free IGF-I of total IGF-I (%)^a^ | 0.53 (0.50, 0.57) | 0.52 (0.49, 0.56) | 0.791 | | |
| IGFBP-1 (nmol/L)^a^ | 0.05 (0.05, 0.06) | 0.05 (0.05, 0.06) | 0.713 | | |
| IGFBP-2 (nmol/L)^a^ | 2.54 (2.42, 2.67) | 2.54 (2.42, 2.66) | 0.967 | | |
| IGFBP-3 (nmol/L)^a^ | 133.5 (131.8, 135.3) | 132.4 (130.6, 134.1) | 0.345 | | |
| IGF-II (nmol/L)^a^ | 60.21 (59.05, 61.39) | 59.89 (58.73, 61.06) | 0.701 | | |
|  |  |  |  | | |
| **Sex hormones** |  |  |  | | |
| Testosterone (nmol/L)^a^ | 15.49 (15.01, 15.98) | 15.33 (14.86, 15.81) | 0.651 | | |
| Free testosterone (pmol/L)^a^ | 258.2 (250.8, 265.9) | 250.7 (243.5, 258.1) | 0.161 | | |
| Sex hormone binding globulin (nmol/L)^a^ | 45.16 (43.88, 46.48) | 46.43 (45.11, 47.78) | 0.183 | | |
| IGF, insulin-like growth factor; IGFBP, IGF-binding protein  ^a^Unknown values for some participants (n=108-149) | | | | |  |

| **Supplemental Table 2: Spearman’s rank correlations between IGF axis analytes and sex hormone concentrations** | | | | | | | | | | | | | | | | | | | |  |
| --- | --- | --- | --- | --- | --- | --- | --- | --- | --- | --- | --- | --- | --- | --- | --- | --- | --- | --- | --- | --- |
|  | **Free IGF-1** | **P value** | **Total IGF-1** | **P value** | **IGFBP-1** | **P value** | **IGFBP-2** | **P value** | **IGFBP-3** | **P value** | **IGF-II** | **P value** | **Ratio of total IGF-I to IGFBP-3** | **P value** | **Testosterone** | **P value** | **Free testosterone** | **P value** | **SHBG** | |
| **Overall (n=1426)**^a^ |  |  |  |  |  |  |  |  |  |  |  |  |  |  |  |  |  |  |  | |
| **Free IGF-1** | 1.000 |  |  |  |  |  |  |  |  |  |  |  |  |  |  |  |  |  |  | |
| **Total IGF-1** | 0.230 | <0.001 | 1.000 |  |  |  |  |  |  |  |  |  |  |  |  |  |  |  |  | |
| **IGFBP-1** | -0.030 | 0.262 | -0.195 | <0.001 | 1.000 |  |  |  |  |  |  |  |  |  |  |  |  |  |  | |
| **IGFBP-2** | -0.074 | 0.005 | -0.155 | <0.001 | 0.590 | <0.001 | 1.000 |  |  |  |  |  |  |  |  |  |  |  |  | |
| **IGFBP-3** | -0.001 | 0.951 | 0.529 | <0.001 | -0.261 | <0.001 | -0.307 | <0.001 | 1.000 |  |  |  |  |  |  |  |  |  |  | |
| **IGF-II** | 0.091 | <0.001 | 0.268 | <0.001 | -0.113 | <0.001 | -0.158 | <0.001 | 0.694 | <0.001 | 1.000 |  |  |  |  |  |  |  |  | |
| **Ratio of total IGF-I to IGFBP-3** | 0.250 | <0.001 | 0.676 | <0.001 | 0.004 | 0.874 | 0.084 | 0.002 | -0.197 | <0.001 | -0.279 | <0.001 | 1.000 |  |  |  |  |  |  | |
| **Testosterone** | 0.014 | 0.593 | -0.026 | 0.336 | 0.331 | <0.001 | 0.318 | <0.001 | -0.203 | <0.001 | -0.159 | <0.001 | 0.145 | <0.001 | 1.000 |  |  |  |  | |
| **Free testosterone** | -0.032 | 0.240 | 0.125 | <0.001 | 0.126 | <0.001 | 0.002 | 0.936 | 0.039 | 0.147 | -0.028 | 0.291 | 0.108 | <0.001 | 0.710 | <0.001 | 1.000 |  |  | |
| **SHBG** | 0.047 | 0.079 | -0.163 | <0.001 | 0.357 | <0.001 | 0.470 | <0.001 | -0.346 | <0.001 | -0.194 | <0.001 | 0.107 | <0.001 | 0.641 | <0.001 | -0.027 | 0.319 | 1.000 | |
|  |  |  |  |  |  |  |  |  |  |  |  |  |  |  |  |  |  |  |  | |
| **Controls (n=713)**^a^ |  |  |  |  |  |  |  |  |  |  |  |  |  |  |  |  |  |  |  | |
| **Free IGF-1** | 1.000 |  |  |  |  |  |  |  |  |  |  |  |  |  |  |  |  |  |  | |
| **Total IGF-1** | 0.192 | <0.001 | 1.000 |  |  |  |  |  |  |  |  |  |  |  |  |  |  |  |  | |
| **IGFBP-1** | <0.001 | 0.998 | -0.206 | <0.001 | 1.000 |  |  |  |  |  |  |  |  |  |  |  |  |  |  | |
| **IGFBP-2** | -0.032 | 0.394 | -0.140 | <0.001 | 0.596 | <0.001 | 1.000 |  |  |  |  |  |  |  |  |  |  |  |  | |
| **IGFBP-3** | 0.007 | 0.850 | 0.538 | <0.001 | -0.267 | <0.001 | -0.303 | <0.001 | 1.000 |  |  |  |  |  |  |  |  |  |  | |
| **IGF-II** | 0.106 | 0.005 | 0.282 | <0.001 | -0.106 | 0.005 | -0.150 | <0.001 | 0.701 | <0.001 | 1.000 |  |  |  |  |  |  |  |  | |
| **Ratio of total IGF-I to IGFBP-3** | 0.186 | <0.001 | 0.631 | <0.001 | 0.007 | 0.853 | 0.100 | 0.007 | -0.242 | <0.001 | -0.323 | <0.001 | 1.000 |  |  |  |  |  |  | |
| **Testosterone** | 0.037 | 0.332 | -0.008 | 0.829 | 0.382 | <0.001 | 0.341 | <0.001 | -0.208 | <0.001 | -0.154 | <0.001 | 0.180 | <0.001 | 1.000 |  |  |  |  | |
| **Free testosterone** | -0.018 | 0.630 | 0.123 | 0.001 | 0.150 | <0.001 | 0.041 | 0.280 | 0.036 | 0.343 | -0.023 | 0.545 | 0.111 | 0.003 | 0.721 | <0.001 | 1.000 |  |  | |
| **SHBG** | 0.074 | 0.049 | -0.140 | <0.001 | 0.415 | <0.001 | 0.466 | <0.001 | -0.344 | <0.001 | -0.189 | <0.001 | 0.141 | <0.001 | 0.661 | <0.001 | 0.015 | 0.691 | 1.000 | |
|  |  |  |  |  |  |  |  |  |  |  |  |  |  |  |  |  |  |  |  | |
| **Cases (n=713)**^a^ |  |  |  |  |  |  |  |  |  |  |  |  |  |  |  |  |  |  |  | |
| **Free IGF-1** | 1.000 |  |  |  |  |  |  |  |  |  |  |  |  |  |  |  |  |  |  | |
| **Total IGF-1** | 0.267 | <0.001 | 1.000 |  |  |  |  |  |  |  |  |  |  |  |  |  |  |  |  | |
| **IGFBP-1** | -0.060 | 0.109 | -0.187 | <0.001 | 1.000 |  |  |  |  |  |  |  |  |  |  |  |  |  |  | |
| **IGFBP-2** | -0.116 | 0.002 | -0.169 | <0.001 | 0.585 | <0.001 | 1.000 |  |  |  |  |  |  |  |  |  |  |  |  | |
| **IGFBP-3** | -0.011 | 0.763 | 0.522 | <0.001 | -0.253 | <0.001 | -0.309 | <0.001 | 1.000 |  |  |  |  |  |  |  |  |  |  | |
| **IGF-II** | 0.075 | 0.046 | 0.253 | <0.001 | -0.121 | 0.001 | -0.164 | <0.001 | 0.684 | <0.001 | 1.000 |  |  |  |  |  |  |  |  | |
| **Ratio of total IGF-I to IGFBP-3** | 0.312 | <0.001 | 0.714 | <0.001 | <-0.001 | 0.999 | -0.066 | 0.078 | -0.156 | <0.001 | -0.239 | <0.001 | 1.000 |  |  |  |  |  |  | |
| **Testosterone** | -0.008 | 0.828 | -0.043 | 0.265 | 0.281 | <0.001 | 0.297 | <0.001 | -0.195 | <0.001 | -0.166 | <0.001 | 0.116 | 0.002 | 1.000 |  |  |  |  | |
| **Free testosterone** | -0.045 | 0.234 | 0.123 | 0.001 | 0.102 | 0.007 | -0.036 | 0.346 | 0.043 | 0.266 | -0.036 | 0.346 | 0.106 | 0.006 | 0.701 | <0.001 | 1.000 |  |  | |
| **SHBG** | 0.016 | 0.666 | -0.186 | <0.001 | 0.302 | <0.001 | 0.476 | <0.001 | -0.342 | <0.001 | -0.193 | <0.001 | 0.077 | 0.040 | 0.617 | <0.001 | -0.068 | 0.074 | 1.000 | |
| ^a^missing values on other IGF axis analytes and sex hormones (n=108-149); SHBG, sex hormone binding globulin | | | | | | | | | | | | | | | | | | | |  |

| **Supplemental Table 3: Odds ratios (95% confidence intervals) for circulating free and total IGF-I concentrations in relation to risks for total prostate cancer by fasting duration and body mass index** | | | | | | | | | | |
| --- | --- | --- | --- | --- | --- | --- | --- | --- | --- | --- |
|  |  | Q1 | Q2 | Q3 | Q4 | P for trend |  | Continuous | P value |  |
| **Free IGF-I** | Median (nmol/L) (range) | 0.022 | 0.073 (0.043-0.098) | 0.132 (>0.098-0.188) | 0.319 (>0.188-7.718) |  |  | per 0.1 nmol/L increase |  |  |
|  |  |  |  |  |  |  |  |  |  |  |
| Fasting status |  |  |  |  |  |  |  |  |  |  |
| No | Cases/controls, n | 116/118 | 97/78 | 96/109 | 102/106 |  |  | 411/411 |  |  |
|  | OR (95% CI)^a^ | 1.00 | 1.27 (0.83, 1.92) | 0.90 (0.60, 1.35) | 0.96 (0.64, 1.45) | 0.577 |  | 1.00 (0.98, 1.02) | 0.814 |  |
| Yes | Cases/controls, n | 140/143 | 76/83 | 59/54 | 59/54 |  |  | 334/334 |  |  |
|  | OR (95% CI)^a^ | 1.00 | 0.94 (0.64, 1.38) | 1.11 (0.72, 1.73) | 1.13 (0.72, 1.78) | 0.515 |  | 1.00 (0.98, 1.02) | 0.799 |  |
|  |  |  |  |  |  |  |  |  |  |  |
| Body mass index |  |  |  |  |  |  |  |  |  |  |
| <30 kg/m^2^ | Cases/controls, n | 183/163 | 117/119 | 110/122 | 127/133 |  |  | 537/537 |  |  |
|  | OR (95% CI)^a^ | 1.00 | 0.87 (0.62, 1.21) | 0.79 (0.56, 1.11) | 0.82 (0.57, 1.18) | 0.334 |  | 1.00 (0.98, 1.02) | 0.808 |  |
| ≥30 kg/m^2^ | Cases/controls, n | 13/13 | 7/7 | 5/6 | 9/8 |  |  | 34/34 |  |  |
|  | OR (95% CI)^a^ | 1.00 | 0.96 (0.28, 3.34) | 0.86 (0.21, 3.47) | 1.13 (0.30, 4.20) | 0.834 |  | 1.03 (0.96, 1.10) | 0.468 |  |
| ^a^model conditioned on the matching variables: center, follow-up time, fasting status, age at blood collection and time at blood collection, and adjusted for laboratory batch | | | | | | | | | | |
